# ZEB1-AS1/miR-133a-3p/LPAR3/EGFR axis promotes the progression of thyroid cancer by regulating PI3K/AKT/mTOR pathway

**DOI:** 10.1186/s12935-020-1098-1

**Published:** 2020-03-29

**Authors:** Wu Xia, Wen Jie

**Affiliations:** 1 The Department of Endocrinology, Jing’an District Centre Hospital of Shanghai (Huashan Hospital Fudan University Jing’An Branch), 259 Xikang Road, Jing’an District, Shanghai, 200040 China; 2 Department of Endocrinology and Metabolism, Huashan Hospital, Fudan University, Shanghai, 200040 China

**Keywords:** ZEB1-AS1, miR-133a-3p, LPAR3, EGFR, TC

## Abstract

**Background:**

Thyroid cancer (TC) is a member of common malignant tumors in endocrine system. To develop effective treatment, further comprehension of understanding molecular mechanism in TC is necessary. In this research, we attempted to search the underlying molecular mechanism in TC.

**Methods:**

ZEB1-AS1 expression was analyzed via qRT-PCR analysis. CCK-8, colony formation, flow cytometry and TUNEL assays were used to evaluate TC cell growth. The interaction between miR-133a-3p and LPAR3, EGFR and ZEB1-AS1 was testified through using RNA pull down and luciferase reporter assays.

**Results:**

LPAR3 and EGFR were expressed at high levels in TC tissues and cell lines. Besides, both LPAR3 and EGFR could promote TC cell growth. Later, miR-133a-3p was searched as an upstream gene of LPAR3 and EGFR, and LPAR3 could partially rescue the suppressive effect of miR-133a-3p overexpression on TC progression, whereas the co-transfection of LPAR3 and EGFR completely restored the inhibition. Next, ZEB1-AS1 was confirmed as a sponge of miR-133a-3p. ZEB1-AS1 has a negative correlation with miR-133a-3p and a positive association with LPAR3 and EGFR through ceRNA analysis. Importantly, ZEB1-AS1 boosted the proliferation and suppressed the apoptosis in TC cells. Through restoration assays, we discovered that ZEB1-AS1 regulated LPAR3 and EGFR expression to mediate TC cell proliferation and apoptosis by sponging miR-133a-3p. Further investigation also indicated the oncogenic role of ZEB1-AS1 by mediating PI3K/AKT/mTOR pathway.

**Conclusions:**

ZEB1-AS1 could be an underlying biomarker in TC.
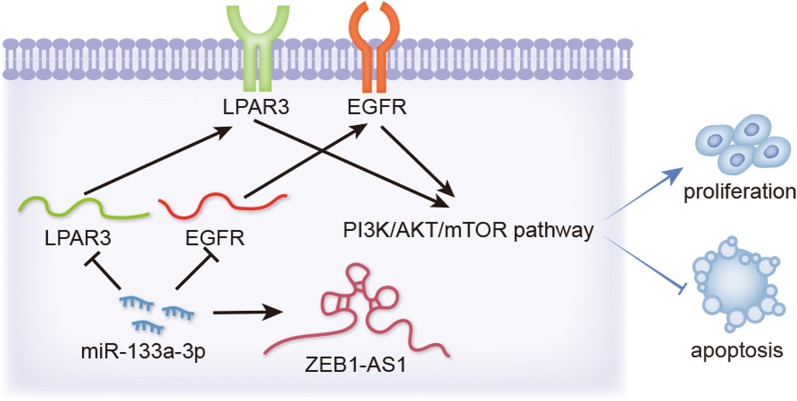

## Background

Thyroid cancer (TC) is a type of cancer in endocrine system and serves as a malignant tumor occurred in the patients [1]. In recent decades, the cases of death associated with TC are continuous to increase [2]. More than 90% of TCs are caused by follicular cells, which are epithelial cells that act on uptaking iodine and synthesizing thyroid hormone [3]. Some TC patients have a favorable prognosis and over 50% of the patients could be curable [4]. Nonetheless, about 30% of patients with invasive TC still suffered from recurrence, distant metastasis, and even death [5]. Hence, it is indispensable to investigate the potential mechanism to develop more effective medical strategies for the patients with TC.

Long non-coding RNAs (lncRNAs) are a group of RNA molecules with a limit in coding proteins and more than 200 nucleotides (nt) in length. Mounting reports have suggested that lncRNAs appear as important regulators in numerous biological processes [6]. MicroRNAs (miRNAs) are a type of short RNAs (20–25 nt) and interact with mRNA 3′-untranslated region (3′-UTR) to degrade the expression of target mRNA or inhibit its translation [7]. Recent research has demonstrated that lncRNAs are mainly distributed in the cytoplasm and act as participants in post-transcriptional regulation through the interaction with potential miRNAs or target mRNAs [8]. Interestingly, increasing evidence has verified that lncRNAs exert regulatory function on tumor progression via ceRNA mechanism, including TC [9, 10]. LncRNA ZEB1-AS1 has been uncovered to be an essential regulator in various tumors, such as osteosarcoma [11], glioma [12], and hepatocellular carcinoma [13]. Additionally, ZEB1-AS1 contributes to prostate cancer progression by inhibiting miR200c and activating ZEB1 [14]. However, the specific performance of ZEB1-AS1 in TC remains obscure.

Lysophosphatidic acid receptor 3 (LPAR3) is the receptor of LPA and has been reported as a regulator in some diseases, such as melanoma [15]. LPAR3 has also been confirmed to be involved in PI3K/AKT pathway in ovarian cancer [16]. Existing evidence has indicated that epidermal growth factor receptor (EGFR) is a vital participant in a variety of cancers, like non-small cell lung cancer [17] and ovarian cancer [18]. In addition, EGFR was widely reported to be implicated in the regulation of PI3K/AKT pathway [19]. More interestingly, LPA elicits rapid transactivation of EGFR by mainly activating LPAR3 in oral squamous carcinoma cells [20].

Here, we aimed to further investigate the biological role and underlying mechanism of LPAR3 and EGFR in TC. The oncogenic role of LPAR3 and EGFR was revealed in TC, and we also found ZEB1-AS1/miR-133a-3p/LPAR3/EGFR axis, which promoted TC progression by regulating PI3K/AKT/mTOR pathway. This discovery could be useful for researching the new therapeutic method for TC patients.

## Methods

### Tissues

TC tissues and paired adjacent non-tumor tissues were acquired from 40 patients at Jing’an District Centre Hospital of Shanghai (Huashan Hospital Fudan University Jing’An Branch) from 2013 to 2018. No patients received treatment of radiotherapy or chemotherapy before surgery. Written informed consents were attained from all subjects. Tissue samples were frozen in liquid nitrogen right after surgery and stored at − 80 °C. The study was approved by the Ethics Committee of Jing’an District Centre Hospital of Shanghai (Huashan Hospital Fudan University Jing’An Branch).

### Cell culture and treatment

Thyroid epithelial cell (Nthy-ori 3-1) and TC cells (SW579, TPC-1, BCPAP and KAT18) were utilized in this study, from the American Type Culture Collection (ATCC; Manassas, VA, USA). Cells were cultivated at 37 °C in RPMI 1640 medium (Invitrogen, Carlsbad, CA, USA) with additional 10% fetal bovine serum (FBS; Gibco, Grand Island, NY, USA) in an incubator with 5% CO_2_. Insulin-like growth factors 1 (IGF-1; Sigma-Aldrich, St. Louis, MO, USA) was applied as the PI3K/Akt signaling pathway activator to treat TPC-1 and BCPAP cells.

### Cell transfection

Specific shRNAs against LPAR3 (sh-LPAR3#1/2), EGFR (sh-EGFR#1/2) or ZEB1-AS1 (sh-ZEB1-AS1#1/2) and their corresponding NC (sh-NC), together with the pcDNA3.1/LPAR3, pcDNA3.1/EGFR and the empty vectors as control (termed as vector), were constructed by Genechem (Shanghai, China). Moreover, miR-133a-3p mimics and NC mimics were formed by GenePharma (Shanghai, China). These plasmids were severally transfected into TPC-1 or BCPAP cells by Lipofectamine 3000 (Invitrogen). As previously described, shRNA sequences were procured for further analysis [21]. The following were shRNA sequences involved in this study: sh-NC (scrambled shRNA): 5′-CCGGTTCTGAAAATTAAAAATTAAACTCGAGTTTAATTTTTAATTTTCAGAATTTTTG-3′, sh-ZEB1-AS1#1: 5′-CCGGGTCAACAATATTAATTTAAGACTCGAGTCTTAAATTAATATTGTTGACTTTTTG-3′, sh-ZEB1-AS1#2: 5′-CCGGGAGGATGAATGCAGATATATACTCGAGTATATATCTGCATTCATCCTCTTTTTG-3′, sh-LPAR3#1: 5′- CCGGAATACATAGGCAATTCCAGCGCTCGAGCGCTGGAATTGCCTATGTATTTTTTTG-3′, sh-LPAR3#2: 5′- CCGGCAGTACATAGAGGATAGTATTCTCGAGAATACTATCCTCTATGTACTGTTTTTG-3′, sh-EGFR#1: 5′-CCGGTAATTCCTCTGCACATAGGTACTCGAGTACCTATGTGCAGAGGAATTATTTTTG-3′, sh-EGFR#2: 5′-CCGGGTGACTTTCTCAGCAACATGTCTCGAGACATGTTGCTGAGAAAGTCACTTTTTG-3′.

### qRT-PCR

TRIzol reagent (Invitrogen) was employed for extraction of total RNA which was later applied to synthesize cDNA via a PrimeScript RT Reagent Kit (Takara, Shiga, Japan). SYBR Green Premix Ex Taq (Takara) on ABI 7900 PCR system (Applied Biosystems, Foster City, CA, USA) was utilized for implementation of qRT-PCR. GAPDH or non-coding RNA U6 was an endogenous control. Values were calculated by 2^−ΔΔCT^.

### CCK-8 assay

TPC-1 or BCPAP cells were put in 96-well plates (2 × 10^3^ cells/well). Cell viability was studied by 10 μL of CCK-8 (DOJINDO, Kumamoto, Japan). Absorbance at 450 nm was recorded via microplate reader (Bio-Rad, Hercules, CA, USA).

### Colony formation assay

TPC-1 or BCPAP cells were planted in 6-well plates (500cells/well) for 2 weeks, followed by fixation for 10 min utilizing 4% paraformaldehyde (PFA; Sigma-Aldrich). Upon this, colonies were subjected to crystal violet (Sigma-Aldrich) for 30 min. In the end, colonies were counted.

### Flow cytometer of apoptosis

TPC-1 or BCPAP cells were washed twice applying PBS (Sigma-Aldrich). Cells were resuspended in 100 µL 1× binding buffer (eBioscience, San Diego, CA, USA). Next, cell suspension was added with Annexin V (Sigma-Aldrich) and propidium iodide (PI; Sigma-Aldrich) in the dark. Cells were subsequently blended with 400 µL 1× binding buffer, followed by analysis via the FACS Calibur flow cytometer (BD Biosciences, San Jose, CA, USA).

### TUNEL assay

Cell apoptosis was explored with the In Situ Cell Death Detection Fluorescein Kit (Roche, Mannheim, Germany). TPC-1 or BCPAP cells on coverslips were immobilized for 1 h using freshly prepared 4% PFA, after which were permeabilized using 0.1% citrate buffer (Sigma-Aldrich) with additional 0.1% Triton X-100 (Sigma-Aldrich). After being rinsed using PBS, cells were incubated for 1 h in TUNEL reaction mix. Coverslips were mounted to a slide upon extensive wash and observed via a fluorescence microscope (Leica, Mannheim, Germany). DAPI (Sigma-Aldrich) was applied in this assay for cell nuclei staining.

### RNA pull-down

Cell lysates of TPC-1 cells were incubated with biotinylated RNA probes including LPAR3 biotin probe, LPAR3 no-biotin probe, EGFR biotin probe and EGFR no-biotin probe, respectively. Upon this, magnetic beads (Invitrogen) were added. Levels of miRNAs were assessed with qRT-PCR.

### Luciferase reporter assay

The wild-type (WT) or mutant (Mut) binding sites of miR-133a-3p to ZEB1-AS1 sequence, 3′-UTR of LPAR3 or EGFR were sub-cloned into pmirGLO dual-luciferase vector (Promega, Madison, WI, USA). The established pmirGLO-LPAR3-WT/Mut, pmirGLO-EGFR-WT/Mut or pmirGLO-ZEB1-AS1-WT/Mut reporter plamsids was co-transfected into TPC-1 or BCPAP cells with miR-133a-3p mimics or NC mimics. The predicted lncRNAs of miR-133a-3p were also sub-cloned into pmirGLO dual-luciferase vector and to co-transfected with miR-133a-3p mimics or NC mimics into cells. Dual luciferase reporter assay system (Promega) was adopted for assessment of luciferase activities.

### Western blot

TPC-1 or BCPAP cells were lysed using RIPA protein extraction regent (Beyotime, Shanghai, China). Protein concentration was evaluated through a BCA protein assay kit (Abcam, Cambridge, USA). Proteins were later separated using 10% SDS-PAGE (Bio-Rad), followed by being shifted to PVDF membranes (Millipore, Billerica, MA, USA). Membranes were probed by use of primary antibodies against LPAR3 (1: 2000 dilution, PA5-27074, Invitrogen), EGFR (1: 2000 dilution, ab52894, Abcam), p-PI3K (1: 1000 dilution, ab182651, Abcam), PI3K (1: 1000 dilution, ab191606, Abcam), p-AKT (1: 1000 dilution, ab131443, Abcam), AKT (1: 10,000 dilution, ab179463, Abcam), p-mTOR (1: 2000 dilution, ab109268, Abcam), mTOR (1: 2000 dilution, ab2732, Abcam) and GAPDH (1: 10,000 dilution, ab245356, Abcam) at 4 °C overnight. After being washed utilizing PBS supplemented with 0.1% Tween-20 (Sigma-Aldrich), membranes were cultivated with HRP-tagged secondary antibodies (1: 10,000 dilution, ab205718, Abcam). Protein bands were assayed by an ECL Substrate Western Blot Detection system (Pierce, Rockford, IL, USA) with the Molecular Imager ChemiDoc XRS system (Bio-Rad).

### Animal study

The nude mice (6-week-old) tested in this research were all obtained from Shi Laike Company (Shanghai, China) and the ethical approval was gained from the Institutional Animal Care and Use Committee of Jing’an District Centre Hospital of Shanghai (Huashan Hospital Fudan University Jing’An Branch). The nude mice were subcutaneously injected with 1 × 10^5^ transfected TPC-1 cells, followed by tumor volumes detected every 4 days. Four weeks later, tumors were acquired from mice for further analysis of volume and weight.

### Immunohistochemistry (IHC) assay

Tumor samples procured from mice were fixed in paraformaldehyde at first. Then they were dehydrated in ethanol solutions, inset in paraffin and cut into 4-μm thickness. Later on, these sections were cultured with primary antibodies against Ki67 (1: 1000 dilution, ab92742, Abcam) and PCNA (1: 1000 dilution, ab92552, Abcam) for a whole night at 4 °C, followed by cultivation with HRP-conjugated secondary antibodies (1: 10,000 dilution, ab205718, Abcam). Finally, they were visualized via microscope (Olympus).

### Statistical analysis

Each experiment was done thrice. Data were shown as mean ± SD. Statistical analysis was carried out using SPSS 18.0 software (IBM, Chicago, IL, USA). Differences in groups were analyzed utilizing Student’s t-test or one-way ANOVA, with the p < 0.05 as significance. Pearson’s method was employed for detecting the correlations among LPAR3, miR-133a-3p, EGFR and ZEB1-AS1 expressions. Overall survival was measured via Kaplan–Meier method with log-rank test.

## Results

### LPAR3 and EGFR are overexpressed in TC tissues and cells

**Fig. 1:**
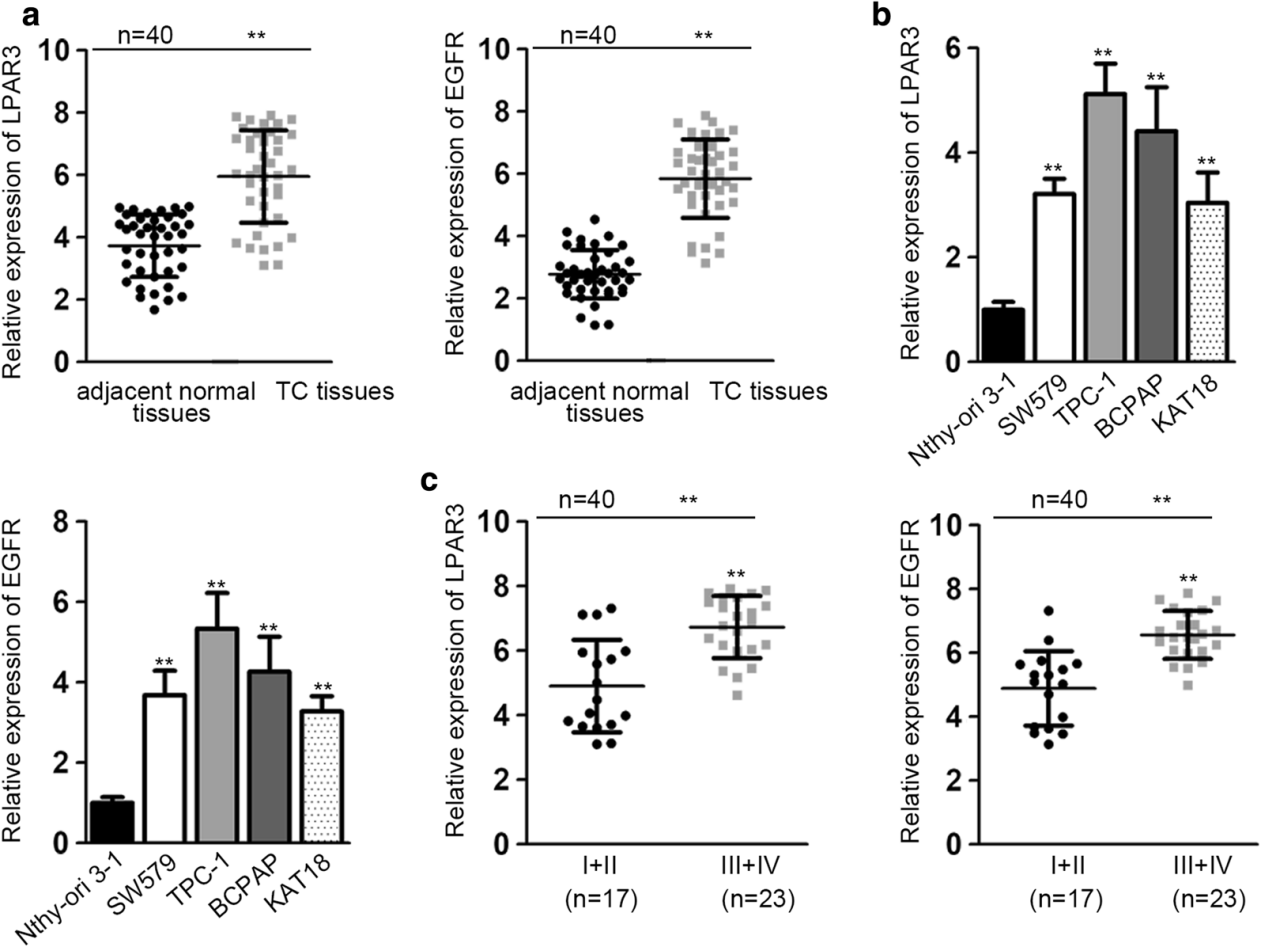
LPAR3 and EGFR are overexpressed in TC tissues and cells. **a** LPAR3 and EGFR expression in TC tissues and paired normal tissues were determined. **b** LPAR3 and EGFR expression in TC cells (SW579, TPC-1, BCPAP, KAT18) and normal cell line (Nthy-ori 3-1) was analyzed by qRT-PCR. **c** The correlation between LPAR3/EGFR expression and pathological stage was presented. **P < 0.01

Through qRT-PCR assay, we observed that the expression of LPAR3 and EGFR was markedly higher in TC tissues than that in matched adjacent tissues (Fig. 1a). To further investigate the expression levels of LPAR3 and EGFR, TC cell lines (SW579, TPC-1, BCPAP, KAT18) were employed with the thyroid epithelial cell line (Nthy-ori 3-1) as a negative reference. Similarly, LPAR3 and EGFR expressions were overexpressed in TC cell lines (Fig. 1b). Moreover, the expression of LPAR3 and EGFR was significantly elevated in TC patients at advanced stage in contrast to those at early stage (Fig. 1c). These results implied that LPAR3 and EGFR were overexpressed in TC tissues and cells.

### LPAR3 and EGFR mediate TC progression

**Fig. 2:**
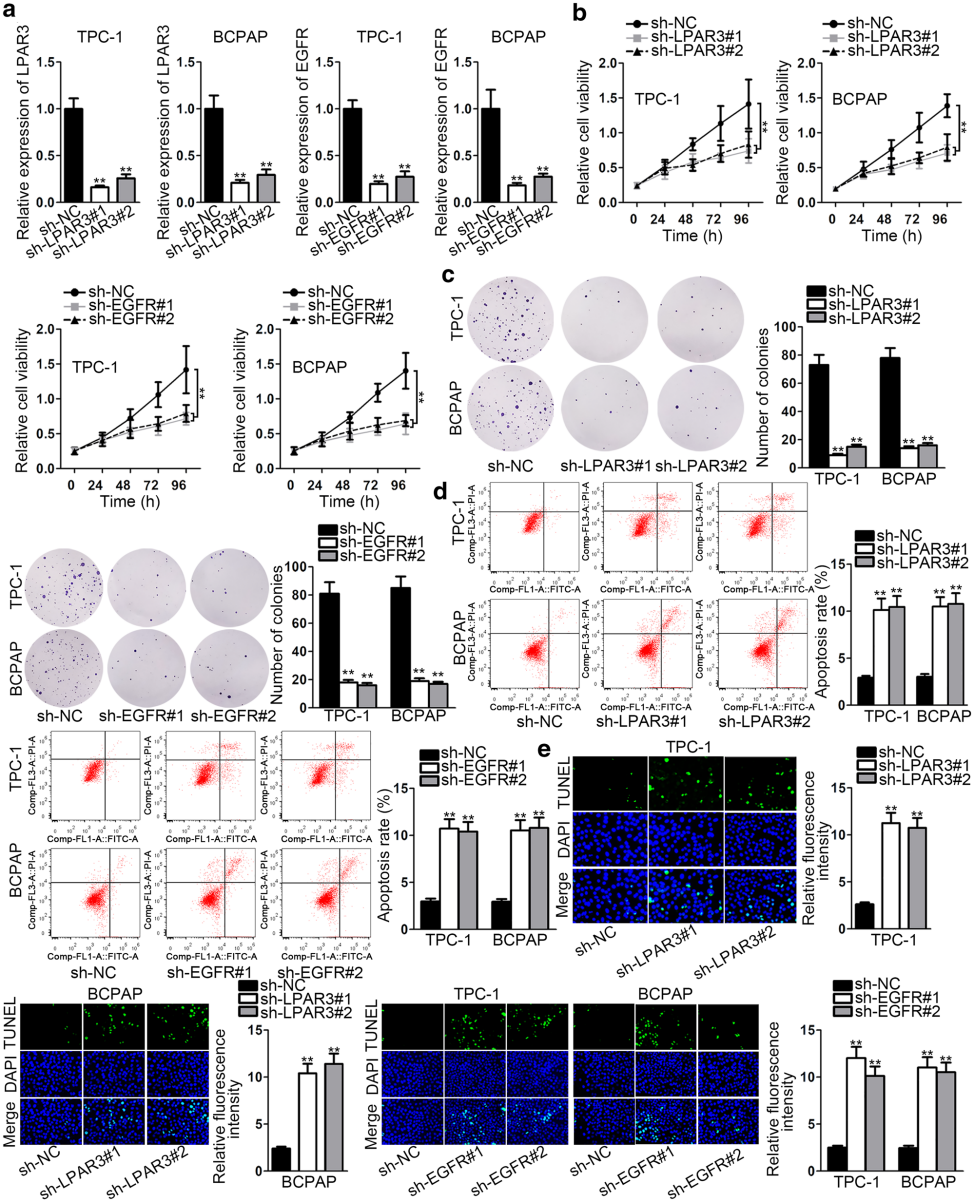
LPAR3 and EGFR mediate TC progression. **a** The interference efficiency of shRNAs specifically targeting to LPAR3 and EGFR (sh-LPAR3#1/2 and sh-EGFR#1/2) were validated by qRT-PCR. Sh-NC was a negative control. **b**, **c** The proliferation of transfected cells was analyzed through CCK-8 and colony formation assays. **d**, **e** Flow cytometry and TUNEL analyses were applied to determine the apoptotic rate of TPC-1 and BCPAP cells after transfection. **P < 0.01

To probe the biological function of LPAR3 and EGFR in TC progression, cell proliferation and apoptosis were analyzed in TPC-1 and BCPAP cells after transfection with shRNAs. Firstly, the expression of LPAR3 and EGFR was markedly lowered by respectively transfecting with sh-LPAR3#1/2 and sh-EGFR#1/2 (Fig. 2a). Followed by LPAR3 or EGFR knockdown, the proliferation ability of TPC-1 and BCPAP cells were analyzed by using CCK-8 and colony formation assays. Results demonstrated that depletion of LPAR3 or EGFR considerably attenuated the proliferation ability of TPC-1 and BCPAP cells (Fig. 2b, c). In addition, flow cytometry and TUNEL analyses were applied to examine the apoptosis ability of TPC-1 and BCPAP cells. It was discovered that apoptosis cells were significantly increased after knocking down LPAR3 or EGFR (Fig. 2d, e). These data suggested that either LPAR3 or EGFR could enhance cell proliferation and restrain cell apoptosis in TC.

### MiR-133a-3p directly targets LPAR3 and EGFR

**Fig. 3:**
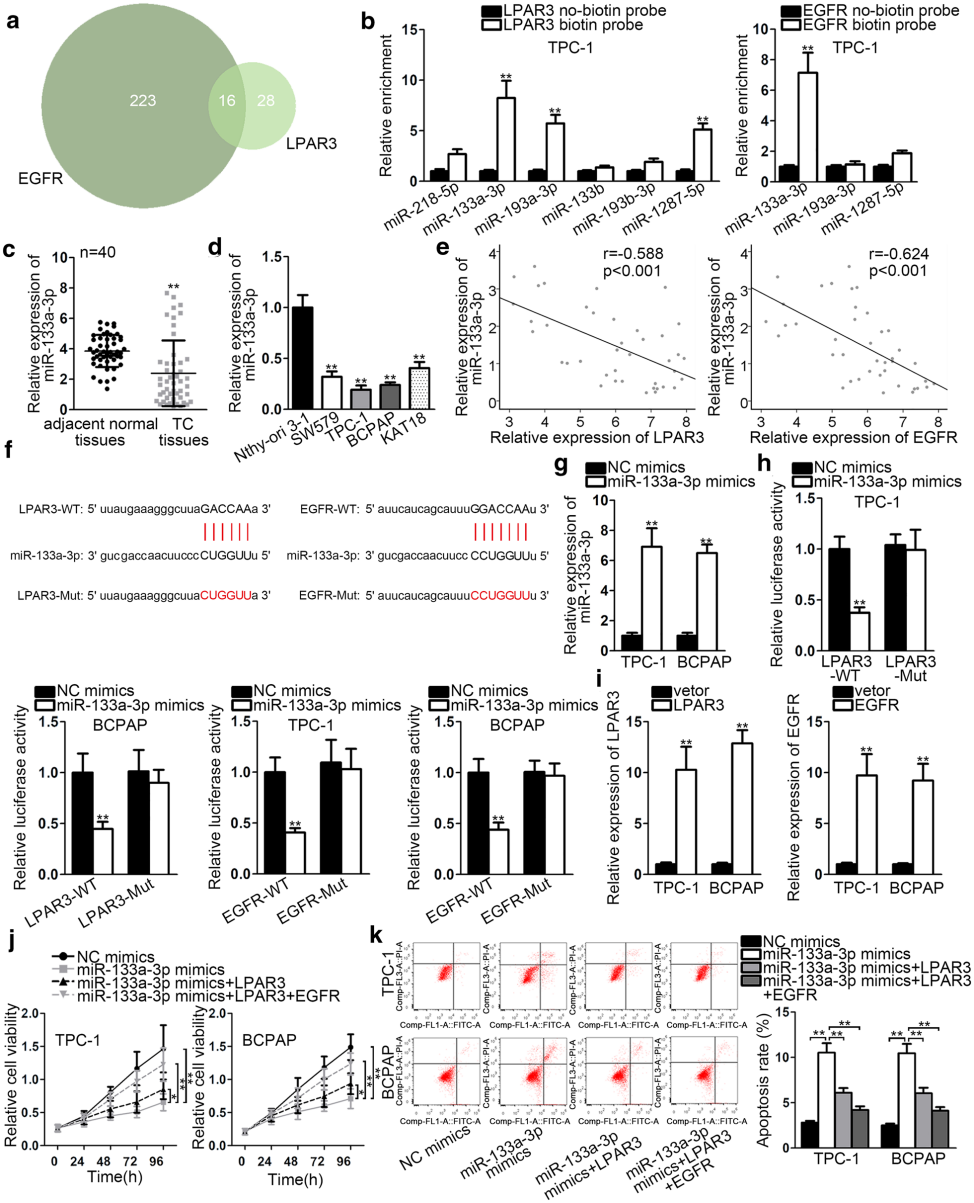
MiR-133a-3p directly targets LPAR3 and EGFR. **a** Venn diagram illustrated the potential miRNAs for LPAR3 and EGFR. **b** RNA pull down assay revealed the possible miRNAs for LPAR3 and EGFR. **c** MiR-133a-3p expression in TC tissues and matched normal tissues was detected via qRT-PCR. **d** MiR-133a-3p expression in TC cell lines and normal cell line was examined via qRT-PCR. **e** The association between miR-133a-3p and LPAR3 or EGFR. **f** MiR-133a-3p was presented to have binding sites on LPAR3 or EGFR. **g** qRT‐PCR was applied to test miR-133a-3p expression in cells transfected with miR-133a-3p mimics or NC mimics. **h** The interaction between miR-133a-3p and LPAR3 or EGFR was confirmed by luciferase reporter assay. **i** LPAR3 or EGFR expression in transfected cells was detected by qRT-PCR. **j** CCK-8 assay was conducted to detect the effect of indicated vectors on cell proliferation. **k** Flow cytometry analysis was utilized to assess the effect of indicated vectors on cell apoptosis. *P < 0.05, **P < 0.01, ***P < 0.001

As the description of ceRNA hypothesis, RNA transcripts interacted with each other by using miRNA response elements (MREs) [22]. Hence, we attempted to search the upstream miRNAs of LPAR3 and EGFR. As shown in Venn diagram, 16 potential miRNAs predicted to bind with both LPAR3 and EGFR were discovered (Fig. 3a). According to dbDEMC website, 6 miRNAs (miR-218-5p, miR-133a-3p, miR-193a-3p, miR-133b, miR-193b-3p and miR-1287-5p) were downregulated in TC. Then, these miRNAs were used for the subsequent research. As shown in Fig. 3b, only miR-133a-3p was significantly enriched by LPAR3 and EGFR biotin probe with RNA pull down assay. Next, miR-133a-3p expression in TC tissues and cells was detected by qRT-PCR analysis. The results displayed a low expression of miR-133a-3p in TC tissues and cells (Fig. 3c, d). Besides, miR-133a-3p illustrated a negative correlation with LPAR3 and EGFR in TC tissues by Pearson analysis (Fig. 3e). Through bioinformatics software (Starbase v3.0), miR-133a-3p was noticed to have binding sequences with LPAR3 and EGFR (Fig. 3f). Subsequently, miR-133a-3p expression was elevated with the transfection of miR-133a-3p mimics (Fig. 3g). Luciferase reporter assay depicted that miR-133a-3p overexpression lessened the luciferase activity of LPAR3-WT and EGFR-WT but that of LPAR3-Mut and EGFR-Mut demonstrated no changes (Fig. 3h). In addition, the expression of LPAR3 and EGFR was upregulated in TPC-1 and BCPAP cells by transfecting with overexpression plasmids (Fig. 3i). Restoration assays implied that the repressive proliferation caused by miR-133a-3p mimics was partially restored by LPAR3 overexpression, whereas the co-transfection of LPAR3 and EGFR could completely recover the effect of miR-133a-3p mimics on cell proliferation (Fig. 3j). Cell apoptosis facilitated by overexpression of miR-133a-3p was partially counteracted with LPAR3 and completely rescued in cells co-transfected with LPAR3 and EGFR (Fig. 3k). Above results validated that miR-133a-3p could directly target LPAR3 and EGFR in TC.

### ZEB1-AS1 sponges miR-133a-3p and regulates LPAR3 and EGFR expression in TC progression

**Fig. 4:**
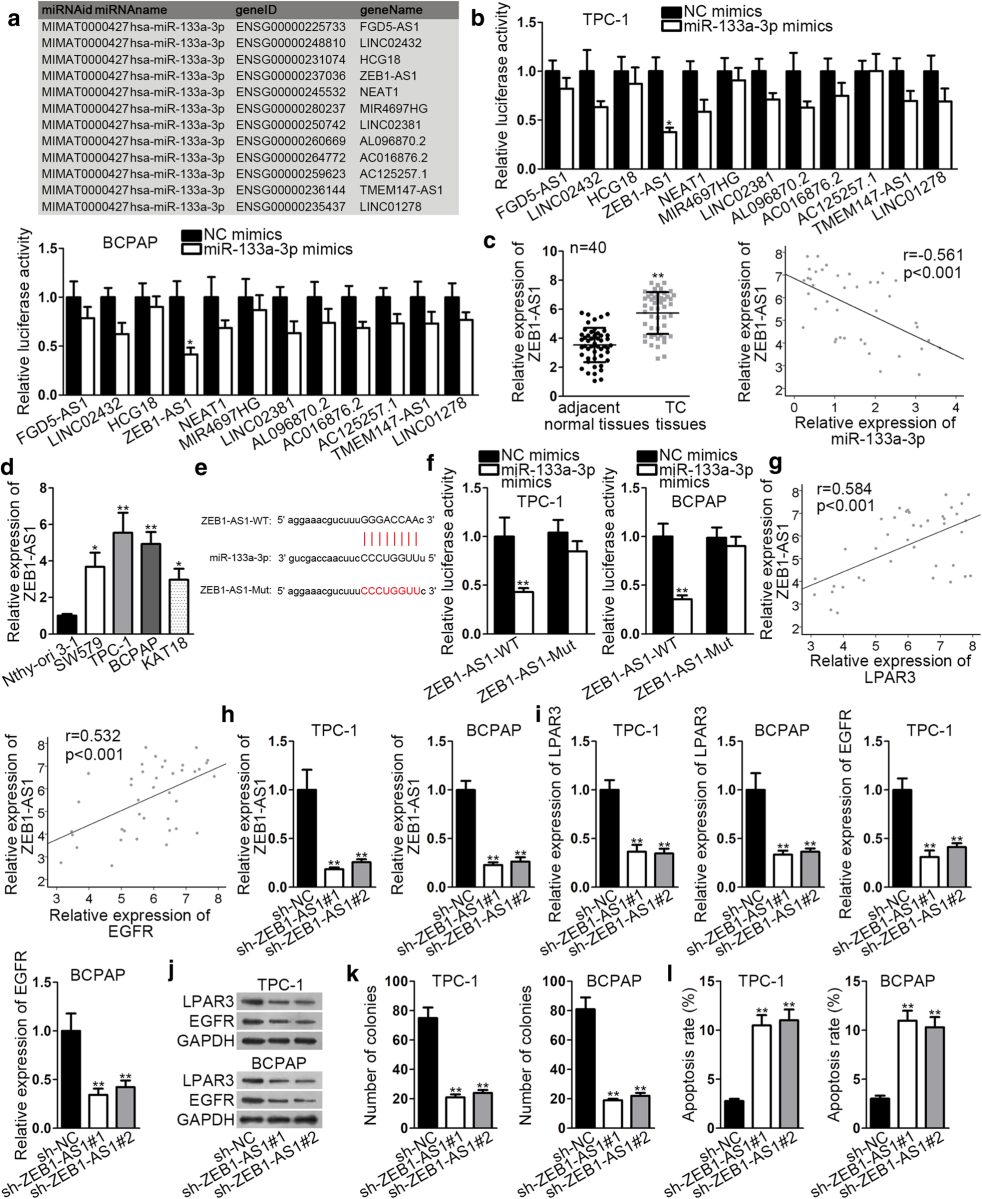
ZEB1-AS1 sponges miR-133a-3p. **a** Potential lncRNAs targeting miR-133a-3p were illustrated. **b** Luciferase reporter assay was employed to screen out the lncRNAs for miR-133a-3p. **c** ZEB1-AS1 expression in TC tissues and the relevance of ZEB1-AS1 with miR-133a-3p were analyzed. **d** ZEB1-AS1 expression in TC cell lines and normal cell line was examined through qRT-PCR. **e** The binding site between ZEB1-AS1 and miR-133a-3p was predicted by starBase. **f** Luciferase reporter assay validated the combination between ZEB1-AS1 and miR-133a-3p. **g** A positive relationship between ZEB1-AS1 and LPAR3/EGFR was revealed. **h** ZEB1-AS1 expression in sh-ZEB1-AS1#1/2-transfected cells was detected via qRT-PCR. Sh-NC was a negative control. **i**, **j** The mRNA and protein expression levels of LPAR3 or EGFR were examined through qRT-PCR and western blot analyses in TPC-1 and BCPAP cells transfected with sh-ZEB1-AS1#1/2 or sh-NC. **k** The effect of ZEB1-AS1 silence on cell proliferation was examined via colony formation. **l** Cell apoptosis was evaluated in transfected cells via flow cytometry analysis. *P < 0.05, **P < 0.01, ***P < 0.001

Accumulating evidences have validated that lncRNAs could inhibit the expression and activity of miRNAs [23]. Here, we aimed to find the underlying lncRNAs for miR-133a-3p. On the basis of ceRNA analysis, starbase was utilized to predict the potential lncRNA. As a result, 12 lncRNAs were screened (Fig. 4a). From luciferase reporter assay, we observed that the luciferase activity of ZEB1-AS1 was reduced by miR-133a-3p mimics while no difference was noticed in other lncRNAs (Fig. 4b). ZEB1-AS1 was much overexpressed in TC tissues and a notable negative association was presented between ZEB1-AS1 and miR-133a-3p (Fig. 4c). Later, upregulated ZEB1-AS1 was also observed in TC cells (Fig. 4d). ZEB1-AS1 was predicted to have a binding sequence in miR-133a-3p (Fig. 4e). The luciferase activity of ZEB1-AS1-WT was obviously attenuated by miR-133a-3p mimics whereas no statistical change was experienced in that of ZEB1-AS1-Mut (Fig. 4f). These results indicated that ZEB1-AS1 was a sponge of miR-133a-3p. In addition, we found a positive relationship between ZEB1-AS1 and LPAR3/EGFR (Fig. 4g). Subsequently, through qRT-PCR analysis, we observed that LPAR3 depletion or EGFR knockdown caused no significant change of ZEB1-AS1 expression in TPC-1 and BCPAP cells (Additional file 1: Fig. S1A, B). However, after we decreased ZEB1-AS1 expression in TPC-1 and BCPAP cells by transfection with sh-ZEB1-AS1#1/2 (Fig. 4h, Additional file 1: Fig. S1C), the expression of LPAR3 and EGFR was observably diminished (Fig. 4i, j, Additional file 2: Fig. S2A). To test the biological effect of ZEB1-AS1 on TC cell proliferation and apoptosis, loss-of-function assays were applied again. Colony formation assay indicated the suppressive role of ZEB1-AS1 deficiency in the proliferation ability of TPC-1 and BCPAP cells (Fig. 4k). Conversely, flow cytometry analysis suggested the promoting effect of ZEB1-AS1 knockdown on cell apoptosis (Fig. 4l). However, overexpression of ZEB1-AS1 could not rescue the suppressive role of silenced LPAR3/EGFR on TC progression, further revealing that ZEB1-AS1 was the upstream gene of LPAR3/EGFR. As illustrated in Additional file 3: Fig. S3A–C, after we elevated the expression of ZEB1-AS1 in TPC-1 cells, the restraining effect of LPAR3/EGFR depletion on cell proliferation was not restored. Likewise, upregulation of ZEB1-AS1 could not rescue the facilitating effect of LPAR3/EGFR deficiency on cell apoptosis (Additional file 3: Fig. S3D, E). To conclude, ZEB1-AS1 sponged miR-133a-3p and indirectly regulated LPAR3 and EGFR expression in TC.

### ZEB1-AS1 enhances TC progression by upregulating LPAR3 and EGFR

**Fig. 5:**
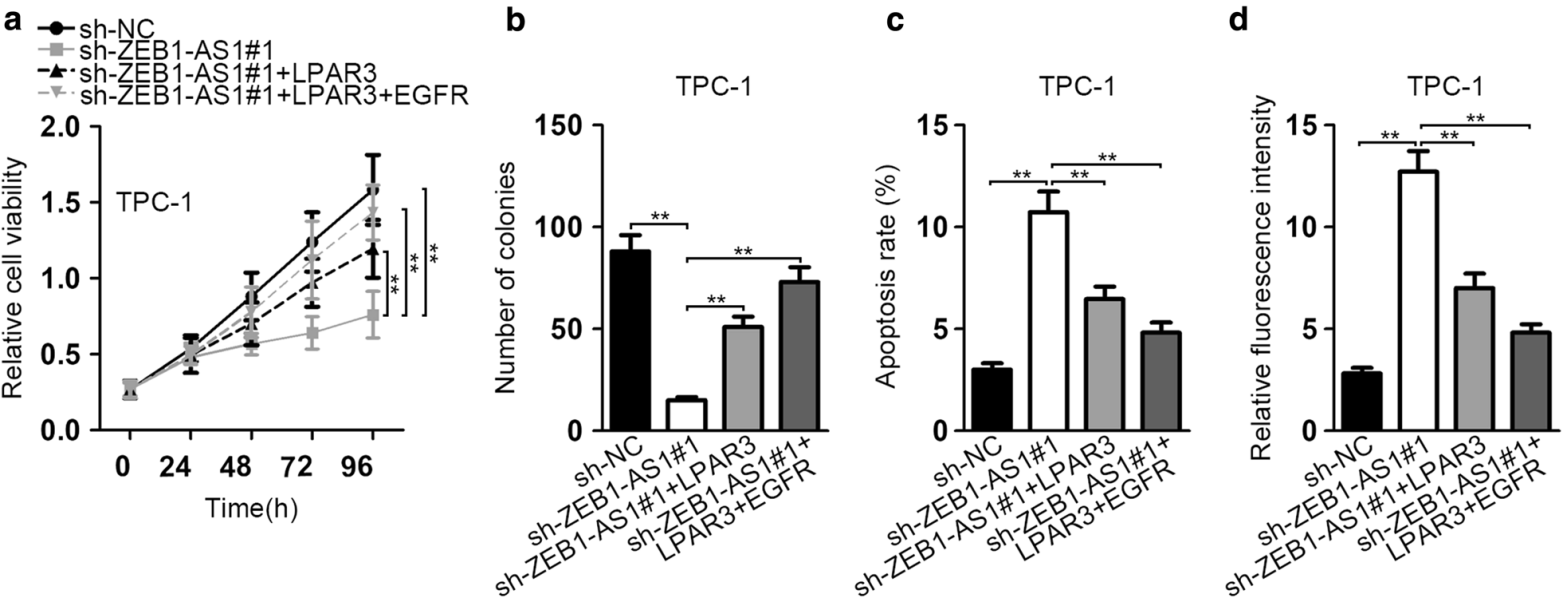
ZEB1-AS1 enhances TC progression by upregulating LPAR3 and EGFR. **a**, **b** The proliferation capability of TPC-1 cells transfected with indicated vectors was evaluated by CCK-8 and colony formation assay. **c**, **d** Flow cytometry analysis and TUNEL assay were employed to measure the apoptosis rate in transfected TPC-1 cells. **P < 0.01

To further confirm whether ZEB1-AS1 facilitated TC development via indirect regulation of LPAR3 and EGFR, some restoration experiments were designed. Cell proliferation capacity repressed by silenced ZEB1-AS1 was partially restored by overexpressed LPAR3, while co-transfection of LPAR3 and EGFR completely countervailed the effect of silenced ZEB1-AS1 on cell proliferation (Fig. 5a, b). Similarly, LPAR3 overexpression partially restored the ZEB1-AS1 silencing-mediated effect on cell apoptosis whereas co-transfection of LPAR3 and EGFR entirely offset the promoting influence on cell apoptosis resulted from ZEB1-AS1 silence (Fig. 5c, d). All outcomes manifested that ZEB1-AS1 enhanced TC progression by upregulating LPAR3 and EGFR.

### ZEB1-AS1 exerts oncogenic effect on TC progression by activating PI3K/AKT/mTOR pathway

**Fig. 6:**
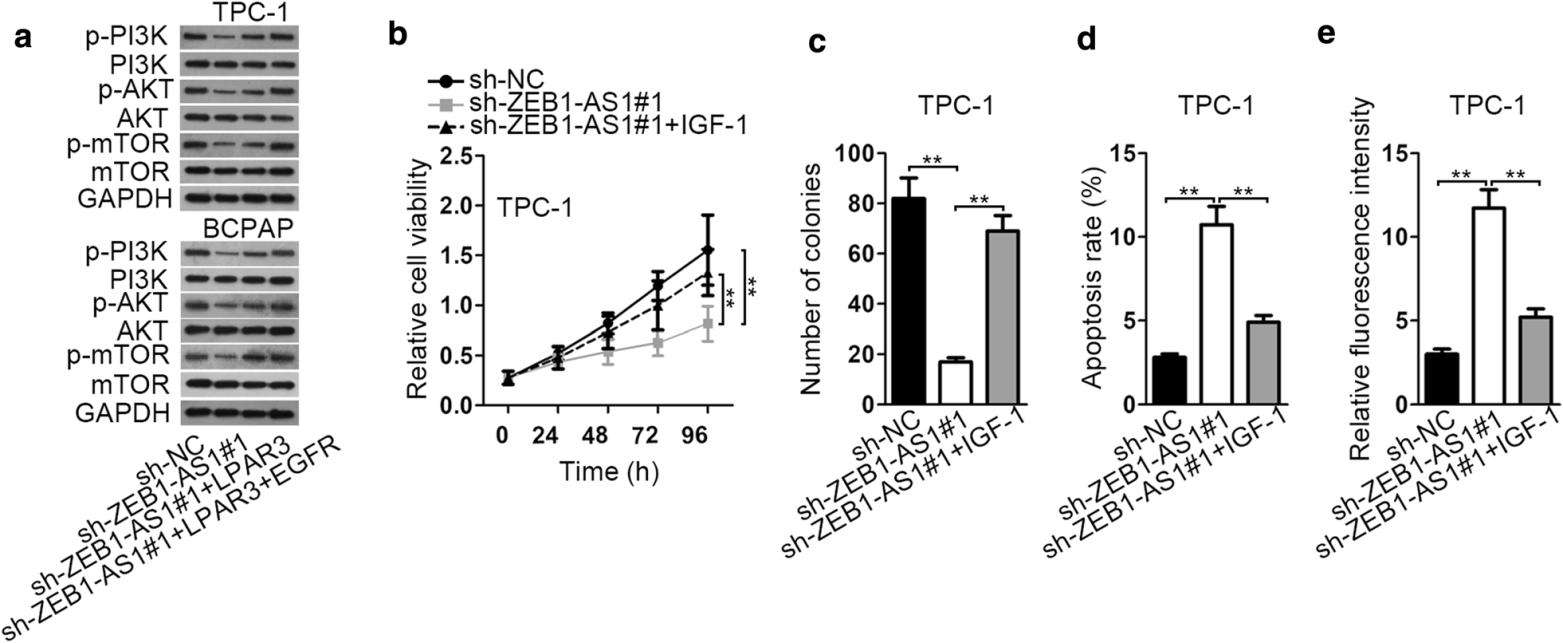
ZEB1-AS1 exerts oncogenic effect on TC progression by activating PI3K/AKT/mTOR pathway. **a** The levels of PI3K, AKT, mTOR and phosphorylated PI3K, AKT, mTOR were tested via western blot analysis in transfected cells. **b**, **c** Cell proliferation in cells transfected with sh-ZEB1-AS1#1 or sh-ZEB1-AS1#1 + IGF-1 was examined via CCK-8 and colony formation assays. Sh-NC was a negative control. **d**, **e** The apoptosis of transfected cells was evaluated via flow cytometry analysis and TUNEL assay. **P < 0.01

Due to the significance of PI3K/AKT/mTOR pathway as well as its close relation to lncRNA-mediated ceRNA mechanism [24, 25], we wondered whether ZEB1-AS1 involved in this pathway. Through western blot assay, the protein levels of PI3K, AKT and mTOR were hardly changed, whereas their phosphorylation levels were remarkably weakened in ZEB1-AS1 silenced cells, and partially rescued by LPAR3 overexpression. Co-transfection of LPAR3 and EGFR effectively restored the suppressive role of ZEB1-AS1 knockdown in PI3K/AKT/mTOR pathway (Fig. 6a, Additional file 2: Fig. S2B). Then, to test whether ZEB1-AS1 regulated TC progression via PI3K/AKT/mTOR pathway, IGF-1, the activator of PI3K/AKT/mTOR pathway [26], was used for the following rescue assays. Prior to rescue assays, IGF-1 expression was uncovered to be upregulated in TC tissues and cell (Additional file 4: Fig. S4A, B). Nevertheless, ZEB1-AS1 upregulation or downregulation had no significant impact on IGF-1 expression (Additional file 4: Fig. S4C, D). Afterwards, addition of IGF-1 reserved the inhibited proliferation resulted from ZEB1-AS1 depletion (Fig. 6b, c). Additionally, the boosted apoptosis in sh-ZEB1-AS1#1-transfected cells was countervailed after treatment of IGF-1 (Fig. 6d, e). Taken together, ZEB1-AS1 exerts oncogenic effect on TC progression through activating PI3K/AKT/mTOR pathway.

### ZEB1-AS1 promotes tumor growth through activation of PI3K/AKT/mTOR pathway

**Fig. 7:**
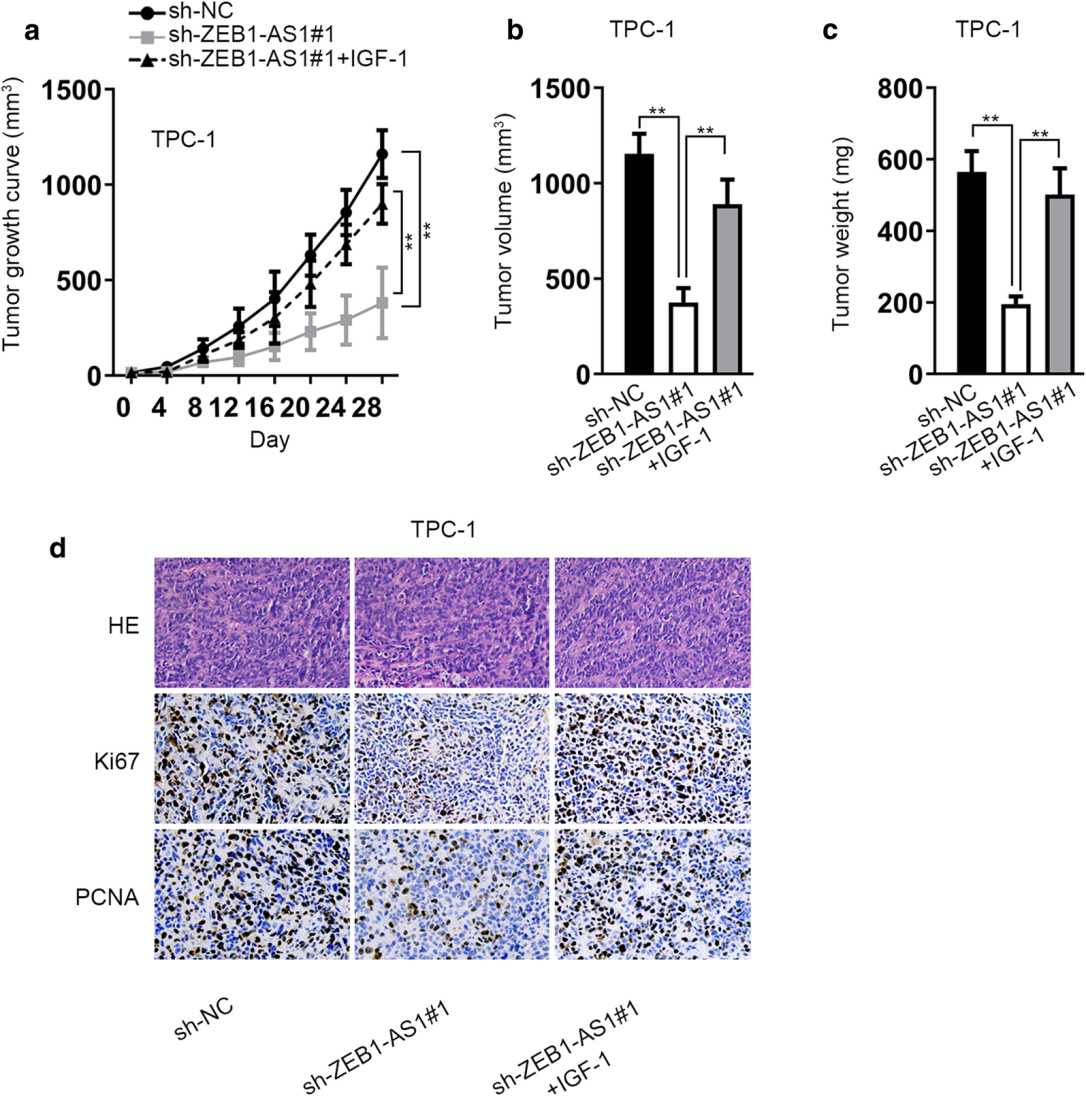
ZEB1-AS1 promotes tumor growth through activation of PI3K/AKT/mTOR pathway. **a**–**c** Tumor growth, volume and weight were analyzed after transfected cells were injected into mice. **d** The expression of proliferation-related proteins (Ki67 and PCNA) was evaluated via IHC analysis. **P < 0.01

To further testify the significant regulation of ZEB1-AS1 on TC tumor growth through PI3K/AKT/mTOR pathway, in vivo assays were conducted. As illustrated in Fig. 7a–c, addition of IGF-1 could recover the suppressive impact of silenced ZEB1-AS1 on tumor growth, volume and weight. Further, IHC analysis of proliferation-associated proteins (Ki67 and PCNA) uncovered that the inhibitive effect of ZEB1-AS1 downregulation on cell proliferation could be reversed by addition of IGF-1 (Fig. 7d). To sum up, ZEB1-AS1 promotes the in vivo tumorigenesis of TC via activation of PI3K/AKT/mTOR pathway.

## Discussion

Former studies have revealed the regulatory function of LPAR3 and EGFR in cancers. For example, LPAR3 acts as a target gene of miR-15b that alleviates tumor growth in ovarian cancer [16]. EGFR was upregulated by FOXK2 in colorectal cancer to enhance the metastasis of colorectal cancer cells [27]. However, the biological effect of LPAR3 and EGFR in TC still needs to be explored. In this study, the overexpressed LPAR3 and EGFR were found in TC tissues and cells. Additionally, LPAR3 and EGFR were disclosed to facilitate cell proliferation and inhibit cell apoptosis in TC. All results indicated the oncogenic property of LPAR3 and EGFR in TC.

In recent years, more and more investigations have implied that miRNA can functionally act as a tumor facilitator or suppressor in diverse diseases, including cancers. Silenced miR-193a-5p sensitizes cells of prostate cancer to docetaxel [28]. MiR-31 promotes EMT in esophageal squamous cell carcinoma through stimulating LATS2 expression [29]. MiR-133a-3p impedes tumor growth and cell metastasis in gastric cancer [30]. MiR-133a-3p boosts prostate cancer progression through activating PI3K/AKT pathway [31]. In this research, low expression of miR-133a-3p was detected in TC tissues and cells. Moreover, miR-133a-3p served as an upstream gene of LPAR3 and EGFR. Further, data from rescue assays revealed that upregulation of LPAR3 and EGFR could rescue the restraining effect of overexpressed miR-133a-3p on TC progression. Briefly, miR-133a-3p served as a tumor-suppressing gene in the regulation of TC development.

LncRNAs played quite complex roles in cell proliferation, apoptosis and differentiation [32, 33]. CeRNA hypothesis that could emerge as a selective function for lncRNAs has been explored in many reports [34]. This critical regulatory mechanism was prevalently identified in the crosstalk among lncRNAs, miRNAs and mRNAs. For instance, lncRNA UICLM functions as a ceRNA in colorectal cancer and promotes its liver metastasis by regulating miRNA-215/ZEB2 axis [9]. LncRNA CRNDE acts as a ceRNA in pancreatic cancer and sponges miR-384 to promote its progression via upregulating IRS1 expression [35]. Our current study uncovered a negative relevance between miR-133a-3p and ZEB1-AS1 and a positive correlation between ZEB1-AS1 and LPAR3/EGFR. In addition, ZEB1-AS1 accelerated cell proliferation and suppressed cell apoptosis in TC. Restoration experiments suggested that LPAR3 partially rescued the inhibitive effect of ZEB1-AS1 knockdown on TC progression, but LPAR3 plus EGFR could substantially restored the effect of silenced ZEB1-AS1 on TC progression. These results depicted that ZEB1-AS1 is involved in ceRNA networks and ZEB1-AS1-miR-133a-3p-LPAR3/EGFR crosstalk played an important role in TC development.

PI3K/AKT/mTOR pathway is commonly accepted as a vital pathway in controlling cancer progression, and lncRNA as ceRNA has been validated to mediate tumorigenesis and development through PI3K/AKT/mTOR pathway [24, 25]. LPAR3 has been manifested to be tightly related to PI3K/AKT pathway in ovarian cancer [16]. Moreover, the close association between EGFR and PI3K/AKT/mTOR pathway has been highlighted in some cancer-related studies [36]. MiR-133a-3p has also been identified to affect the bone metastasis of prostate cancer via the activation of PI3K/AKT pathway [31]. However, whether and how ZEB1-AS1 regulates TC progression as well as the relation of ZEB1-AS1 to PI3K/AKT/mTOR pathway in TC remain obscure, which is worth exploring. In the present study, ZEB1-AS1 knockdown lowered the levels of phosphorylated PI3K, AKT and mTOR, whereas overexpression of LPAR3 and EGFR could recovered the levels. Hence, we speculated that PI3K/AKT/mTOR pathway was involved in TC progression. Through rescue assays, we confirmed that IGF-1, the activator of PI3K/AKT/mTOR pathway [26], could counteract the suppressive influence of ZEB1-AS1 silence on tumor growth and development of TC.

## Conclusion

All these data clarified that ZEB1-AS1/miR-133a-3p/LPAR3/EGFR axis could promote TC development via activating PI3K/AKT/mTOR pathway. This discovery might provide a meaningful revelation for improving the treatment of TC patients.

## Supplementary information

**Additional file 1: Figure S1.** (A, B) The expression of ZEB1-AS1 was examined via qRT-PCR after TPC-1 and BCPAP cells were transfected with different plasmids. (C) The efficiency of ZEB1-AS1 knockdown was evaluated via northern blot, **P < 0.01.

**Additional file 2: Figure S2.** (A, B) Western blot assays of Fig. 4j and Fig. 6a were quantified respectively. **P < 0.01.

**Additional file 3: Figure S3.** (A) The efficiency of ZEB1-AS1 overexpression was evaluated through qRT-PCR. (B, C) The proliferation ability of TPC-1 cells transfected with different plasmids was assessed via CCK-8 and colony formation. (D, E) The apoptosis ability of transfected cells was analyzed via flow cytometry and TUNEL. **P < 0.01. n.s.: no significant.

**Additional file 4: Figure S4.** (A, B) Upregulated IGF-1 was detected in TC tissues and cells via qRT-PCR analysis. (C, D) IGF-1 expression was examined via qRT-PCR after TPC-1 and BCPAP cells were transfected with different plasmids. **P < 0.01.

## Data Availability

Not applicable.

## Abbreviations

lncRNAs: long noncoding RNAs
TC: thyroid cancer
ceRNA: competing endogenous RNA
miRNAs: microRNAs
ZEB1-AS1: ZEB1 antisense RNA 1
LPAR3: lysophosphatidic acid receptor 3
EGFR: epidermal growth factor receptor
FBS: fetal bovine serum
shRNA: short hairpin RNA
NC: negative control
qRT-PCR: quantitative real-time polymerase chain reaction
GAPDH: glyceraldehyde-3-phosphate dehydrogenase
CCK-8: cell counting kit-8
PBS: phosphate buffer saline
PVDF: polyvinylidene fluoride
BCA: bicinchoninic acid
SD: standard deviation
