# Supplementary figures and images for: ZEB1-AS1/miR-133a-3p/LPAR3/EGFR axis promotes the progression of thyroid cancer by regulating PI3K/AKT/mTOR pathway
Source: Cancer Cell Int. 2020 Mar 29;20:94. doi: 10.1186/s12935-020-1098-1 (PMC7103072; doi:10.1186/s12935-020-1098-1)

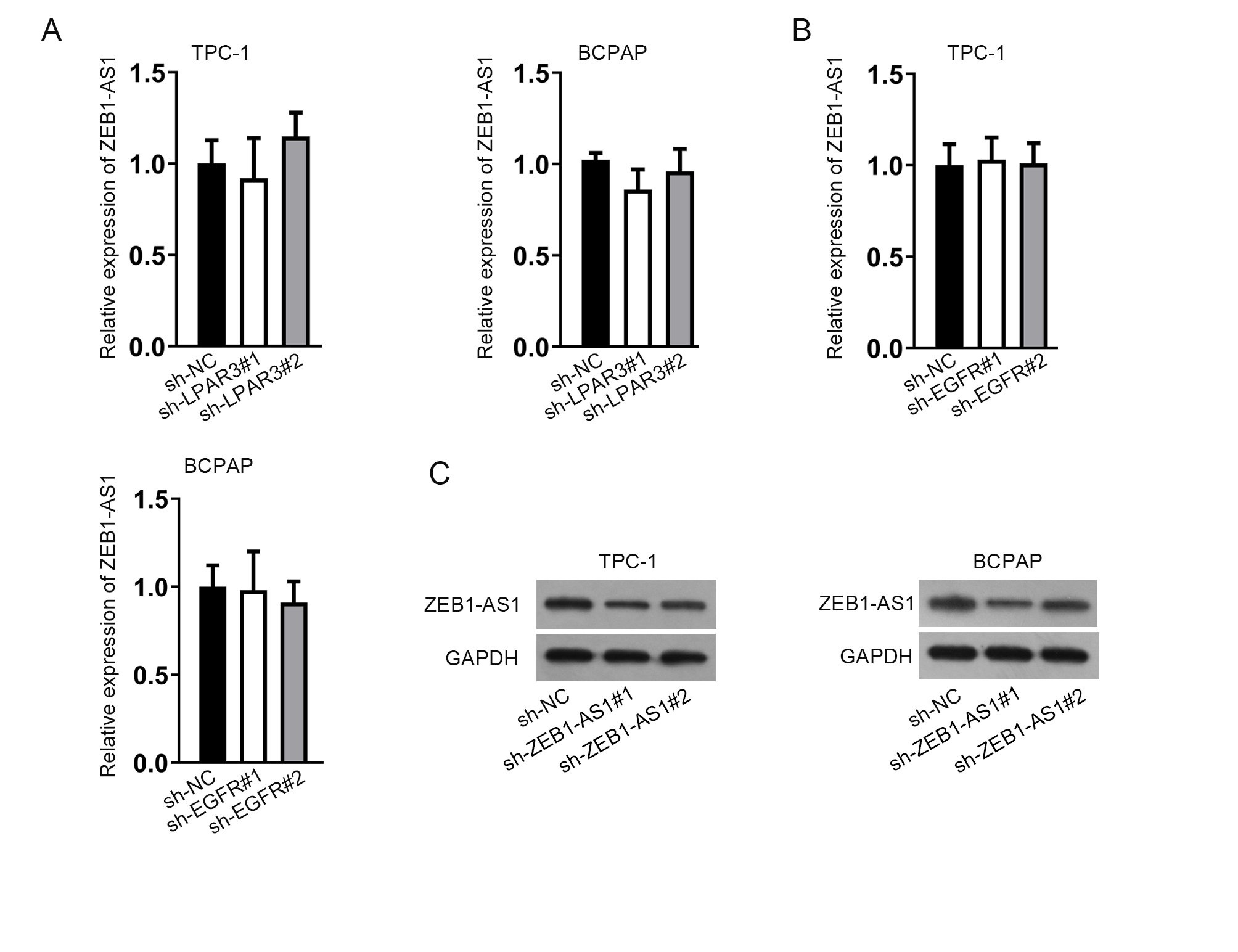

Supplement: Supplementary file 1 — Additional file 1: Figure S1. (A, B) The expression of ZEB1-AS1 was examined via qRT-PCR after TPC-1 and BCPAP cells were transfected with different plasmids. (C) The efficiency of ZEB1-AS1 knockdown was evaluated via northern blot, **P < 0.01. [file 12935_2020_1098_MOESM1_ESM.tif]

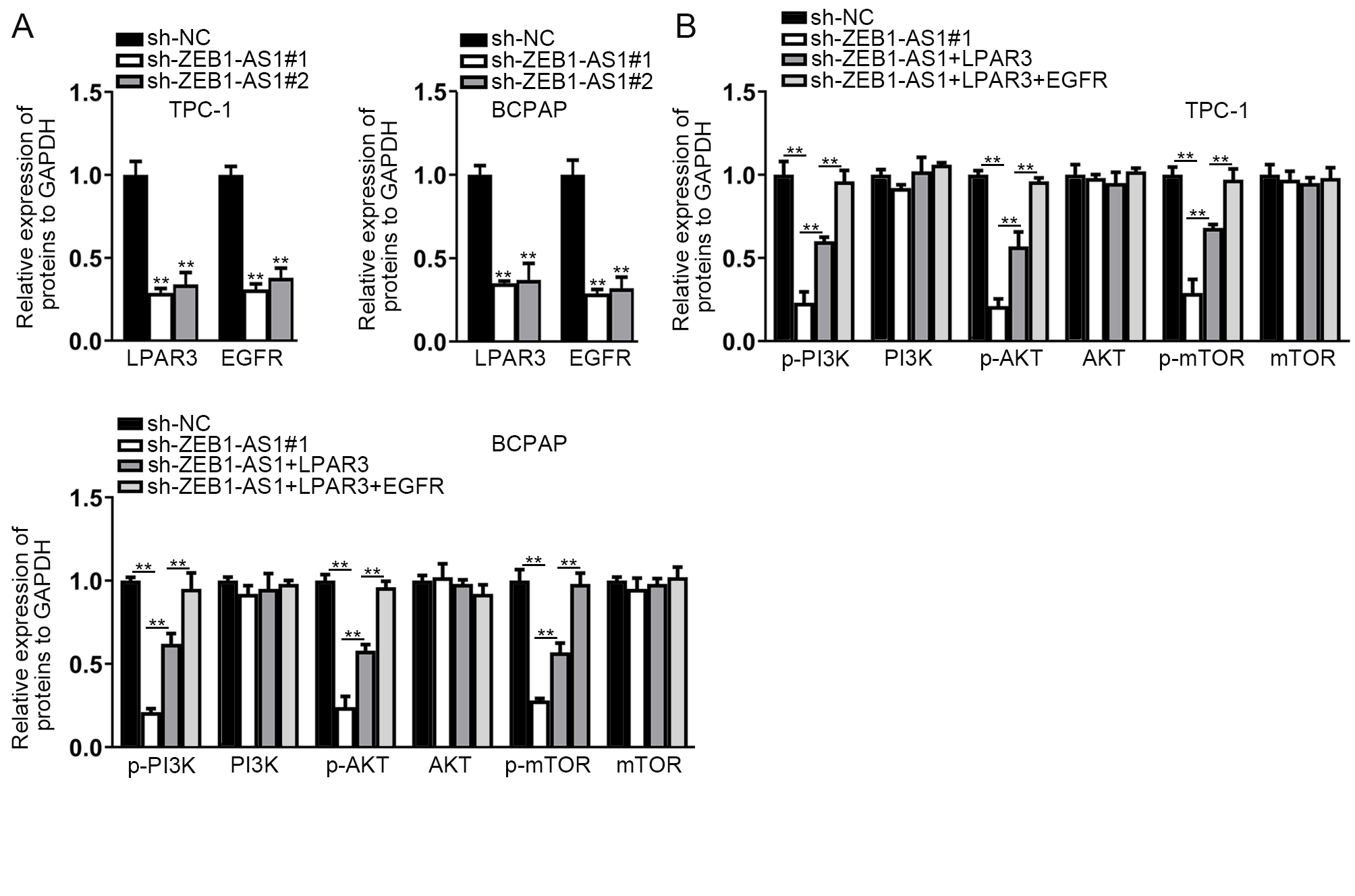

Supplement: Supplementary file 2 — Additional file 2: Figure S2. (A, B) Western blot assays of Fig. 4j and Fig. 6a were quantified respectively. **P < 0.01. [file 12935_2020_1098_MOESM2_ESM.tif]

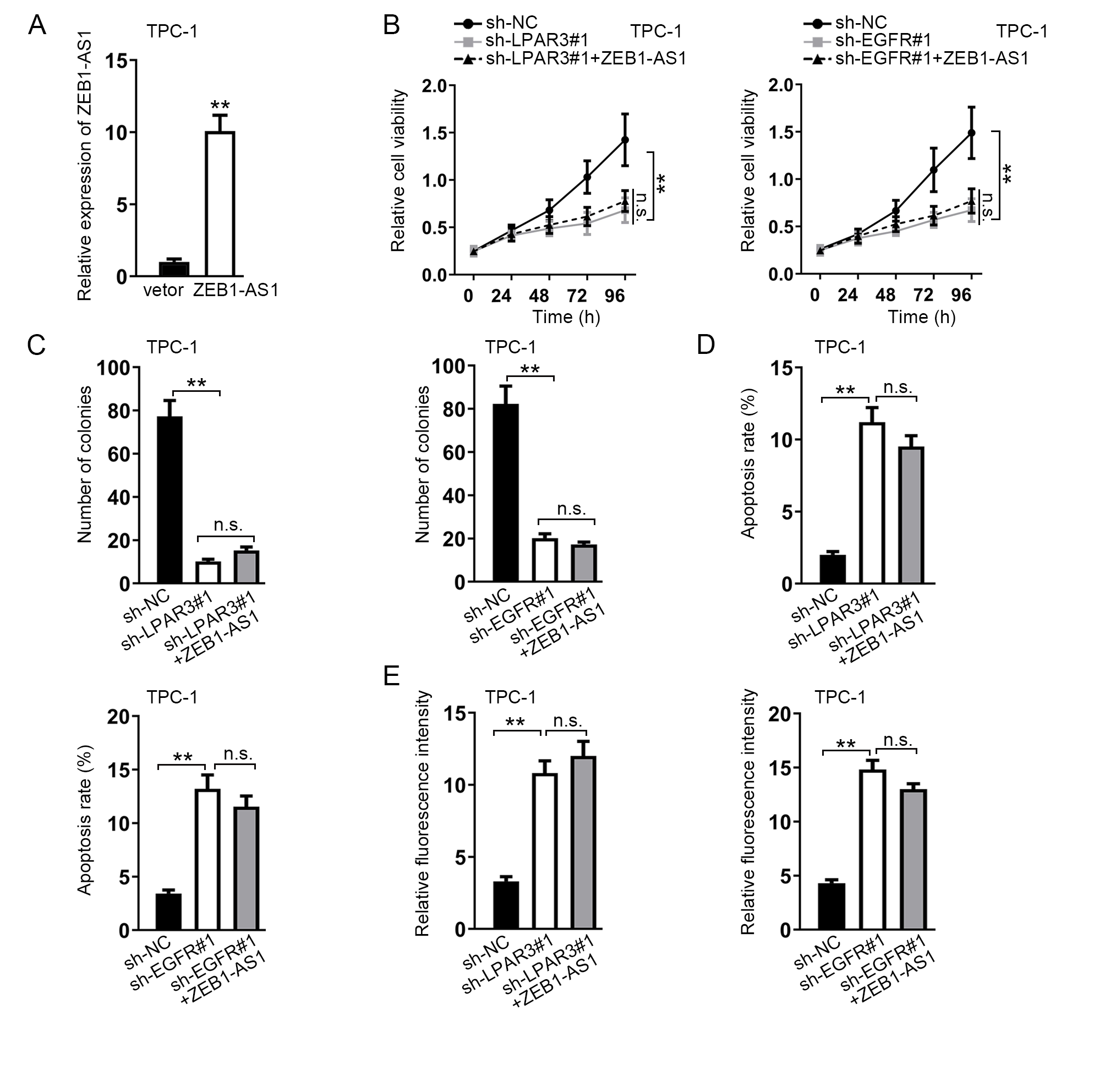

Supplement: Supplementary file 3 — Additional file 3: Figure S3. (A) The efficiency of ZEB1-AS1 overexpression was evaluated through qRT-PCR. (B, C) The proliferation ability of TPC-1 cells transfected with different plasmids was assessed via CCK-8 and colony formation. (D, E) The apoptosis ability of transfected cells was analyzed via flow cytometry and TUNEL. **P < 0.01. n.s.: no significant. [file 12935_2020_1098_MOESM3_ESM.tif]

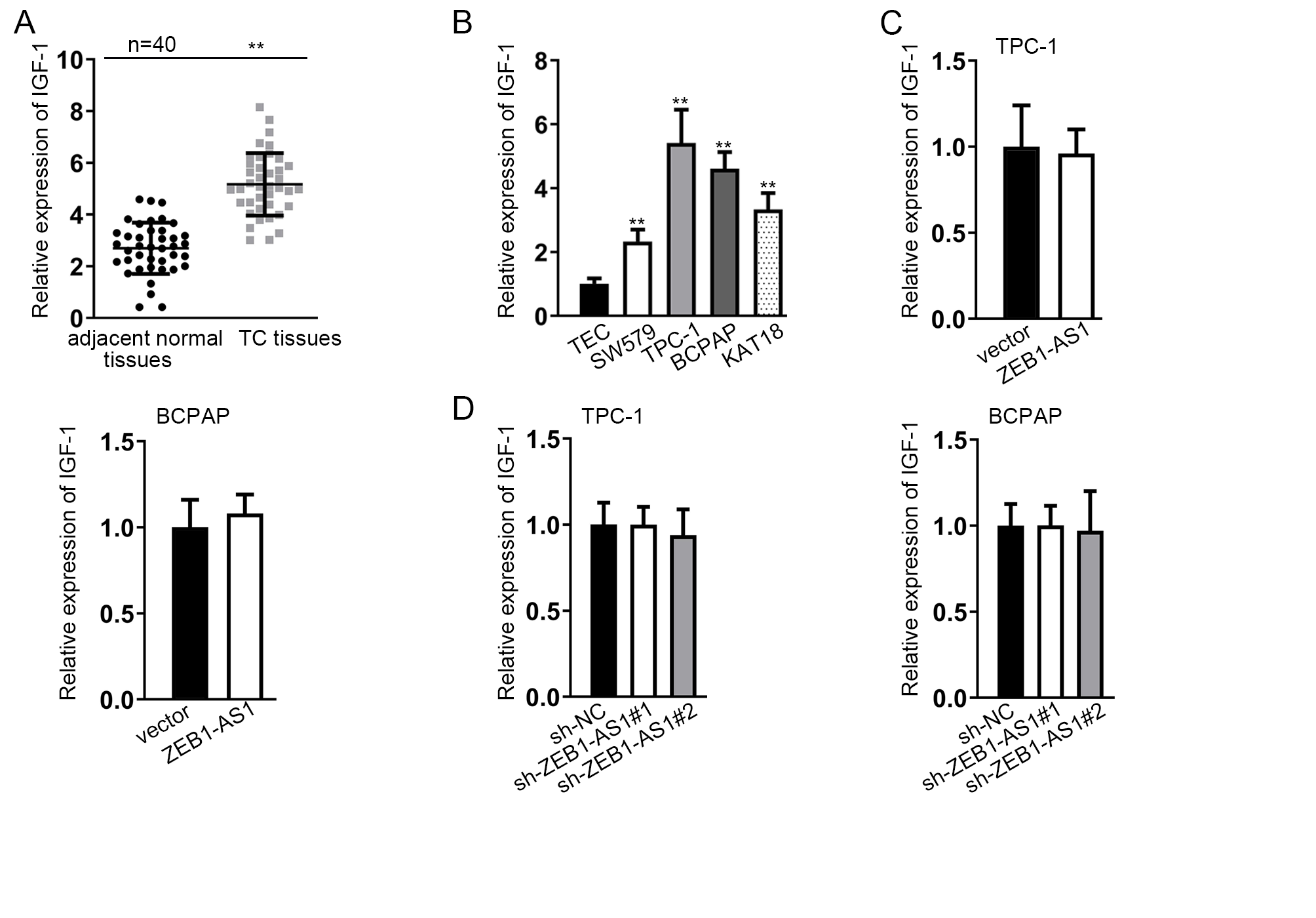

Supplement: Supplementary file 4 — Additional file 4: Figure S4. (A, B) Upregulated IGF-1 was detected in TC tissues and cells via qRT-PCR analysis. (C, D) IGF-1 expression was examined via qRT-PCR after TPC-1 and BCPAP cells were transfected with different plasmids. **P < 0.01. [file 12935_2020_1098_MOESM4_ESM.tif]
